# Impact of maternal anemia during pregnancy on neonatal metabolic profiles: evidence from the Beijing Birth Cohort Study

**DOI:** 10.1186/s12884-025-07626-9

**Published:** 2025-04-26

**Authors:** Shunan Wang, Wei Zheng, Jinqi Zhao, Lulu Li, Yue Tang, Lifei Gong, Lijin Gu, Guanghui Li, Yuanyuan Kong

**Affiliations:** 1 Department of Newborn Screening Center, Beijing Obstetrics and Gynecology Hospital, Capital Medical University, Beijing Maternal and Child Healthcare Hospital, Beijing, China; 2 Division of Endocrinology and Metabolism, Department of Obstetrics, Beijing Obstetrics and Gynecology Hospital, Beijing Maternal and Child Health Care Hospital, Capital Medical University, Beijing, China

**Keywords:** Anemia during pregnancy, Newborn metabolic profiles, Amino acids, Acylcarnitines, Fatty acid oxidation, Metabolism

## Abstract

**Background:**

Anemia during pregnancy is associated with various adverse neonatal outcomes. However, the association between maternal anemia during pregnancy and newborn metabolic profiles remains unclear. This study aimed to investigate whether anemia during pregnancy is associated with alterations in neonatal metabolic profiles.

**Methods:**

This prospective observational cohort study included 12,116 pregnant women, with or without gestational anemia, recruited through the Beijing Birth Cohort Study (ChiCTR2200058395), along with their neonates born between July 2021 and October 2022 in Beijing, China.

**Results:**

Among the 12,116 participants, 576 pregnant women were diagnosed with anemia (Anemia group), while 11,540 did not have anemia (Control group). The rates of metabolic profile abnormalities were significantly higher in the Anemia group compared to the Control group (*P* < 0.05): 20.83% vs. 16.1% for the overall metabolic profile, 11.9% vs. 9.25% for amino acid profiles, and 11.11% vs. 8.04% for acylcarnitine profiles. Individual metabolic indicators showed significant differences: alanine and arginine levels significantly decreased, while tyrosine levels significantly increased in the Anemia group. Notably, most acylcarnitines indicators (C0, C2, C4DC + C5-OH, C5DC + C6-OH, C6, C6DC, C10, C10:1, C12, C12:1, C14, C14:1, C14:2, C16, C16:1, C16:1-OH, C18, and C18:1) were significantly reduced in the Anemia group, except for C5, which was elevated. Pathway analysis revealed that these alterations were associated with beta-oxidation of very long-chain fatty acids, oxidation of branched-chain fatty acids, mitochondrial beta-oxidation of long-chain saturated fatty acids, and fatty acid metabolism. All of these pathways were related to fatty acid oxidation. Sensitive analyses in normal birth weight (NBW) and term infants (TI) confirmed these findings and demonstrated their robustness. In addition, in NBW infants and TIs, citrulline and arginine were significantly decreased, which were associated with aspartate metabolism and the urea cycle.

**Conclusions:**

Maternal anemia during pregnancy is significantly associated with alterations in neonatal metabolic profiles, particularly in fatty acid beta-oxidation and related pathways. These findings highlight the potential metabolic consequences of gestational anemia and provide insights into its role in adverse neonatal outcomes and abnormal newborn screening results.

**Supplementary Information:**

The online version contains supplementary material available at 10.1186/s12884-025-07626-9.

## Introduction

Anemia during pregnancy is defined by the World Health Organization (WHO) as a hemoglobin level < 110 g/L [1]. It is a common complication during pregnancy affecting approximately 56 million pregnant women worldwide [2]. In China, the prevalence of anemia during pregnancy was 30.7% (95%CI: 26.6%, 34.7%) according to a previous study. Based on the WHO classification, this prevalence falls under the category of a “moderate” public health problem (20.0–39.9%) [3].

The primary causes of anemia during pregnancy include decreased hemoglobin and erythrocyte synthesis due to insufficient bioavailability of hematopoietic nutrients and a disproportionate plasma volume relative to red blood cell mass. While such adaptations may enhance maternal survival and promote fetal growth and development, they can compromise optimum placental development and fetal health in the postnatal period [4]. Anemia during pregnancy poses significant risks to both the mother and the offspring [4, 5], being associated with adverse outcomes such as preterm birth, low birth weight [6], low Apgar scores, neonatal anemia [7], and multi-system disorders including neurodevelopmental disorders [8, 9], behavioral problems [10], and respiratory diseases [11]. The impact of anemia in pregnancy on neonatal health should be taken seriously.

The concept of the fetal origin of adult disease suggests that early life conditions can “program” the fetus for a spectrum of adverse health outcomes in adulthood [12]. Previous studies indicate that neonatal metabolism is associated with neurological development later in life [13, 14] Our previous work has revealed significant associations between maternal glucose trajectories throughout pregnancy and neonatal metabolic pathways, including arginine and proline metabolism, the urea cycle, and fatty acid oxidation (FAO) [15]. Additionally, previous studies have demonstrated that newborn metabolic patterns can be influenced by gestational complications [16] such as gestational hypertension [17, 18], maternal lifestyle, and dietary habits. However, the relationship between anemia during pregnancy and newborn metabolic patterns remains unclear. To address this gap, we conducted a prospective study to investigate the effects of anemia during pregnancy on neonatal amino acid (AA) and acylcarnitine (AC) metabolism.

## Methods

### Study design

This study was a prospective observational cohort study, conducted as part of the Beijing Birth Cohort Study (ChiCTR2200058395; Registration date: April 8, 2022). The study was carried out between January 2016 and December 2021 at Beijing Obstetrics and Gynecology Hospital, Capital Medical University. Pregnant women receiving routine prenatal care were enrolled during their antenatal visits, and data were collected without any assignment to interventions or deviation from usual clinical practice.

Maternal anemia during pregnancy was identified based on hemoglobin concentrations measured during routine antenatal check-ups. Neonatal heel blood samples were collected 72 h after exclusive breastfeeding to analyze amino acid (AA) and acylcarnitine (AC) profiles using standard newborn screening protocols.

Women with twin or multiple pregnancies and those with chronic diseases, such as kidney, heart, or liver diseases, were excluded. Additionally, participants without baseline information or neonatal AA and AC profiles were excluded. Further details about the study can be found in a previous publication [19]. The study adhered to the principles of the Declaration of Helsinki and was approved by the Beijing Obstetrics and Gynecology Hospital Ethics Committee (2018-ky−009–01). Written informed consent to participate was obtained from the participants or their legal guardians.

### Study measurements

The primary exposure was anemia during pregnancy, with the diagnosis occurring in the third trimester between 28 and 40 weeks of gestation. The primary outcome was neonatal AA and AC profiles. Anemia during pregnancy is defined by the WHO as a hemoglobin level < 110 g/L [1]. Hemoglobin levels were measured during hospitalization prior to delivery, and anemia was diagnosed if the concentration was less than 110 g/L.

Analyze amino acid (AA) and acylcarnitine (AC) profiles were analyzed using tandem mass spectrometry (MS/MS) with a TQ Acquity Mass Spectrometer (Waters Corporation, United States), a triple quadrupole mass spectrometry system. The NeoBase Non-derivatized MSMS Kit (PerkinElmer, Turku, Finland) was used to detect amino acids and acylcarnitines associated with metabolic disorders. Strict quality control measures ensured accuracy and reliability. The instrument was calibrated daily with standard solutions to maintain mass accuracy within ± 5 ppm. Stable isotope-labeled internal standards were included in all samples to correct signal fluctuations. Each batch contained blank samples, pooled quality control samples from known standards, and patient sample replicates. These were used to assess instrument stability and precision. QC sample peak areas were monitored throughout the analysis. Deviations exceeding 20% triggered reanalysis. Principal component analysis (PCA) was applied to identify batch effects and enhance data consistency.

AA profiles included alanine, arginine, citrulline, glycine, leucine + isoleucine + hydroxyproline, methionine, ornithine, phenylalanine, proline, tyrosine, and valine. AC profiles included free carnitine (C0), acetylcarnitine (C2), propionylcarnitine (C3), malonylcarnitine + 3-hydroxy-butyrylcarnitine (C3DC + C4-OH), butyrylcarnitine (C4), methylmalonylcarnitine + 3-hydroxy-isovalerylcarnitine (C4DC + C5-OH), isovalerylcarnitine (C5), tiglylcarnitine (C5:1), glutarylcarnitine + 3-hydroxyl-hexanoylcarnitine (C5DC + C6-OH), hexanoylcarnitine (C6), 3-methylglutarylcarnitine (C6DC), octanoylcarnitine (C8), succinylacetone (SA), decanoylcarnitine (C10), decenoylcarnitine (C10:1), decadienoylcarnitine (C10:2), dodecanoylcarnitine (C12), dodecenoylcarnitine (C12:1), tetradecanoylcarnitine (C14), 3-hydroxy-tetradecanoylcarnitine (C14-OH), tetradecenoylcarnitine (C14:1), tetradecadienylcarnitine (C14:2), hexadecanoylcarnitine (C16), 3-hydroxyhexadecanoylcarnitine (C16-OH), hexadecenoylcarnitine (C16:1), 3-hydroxylhexadecenoylcarnitine (C16:1-OH), octadecanoylcarnitine (C18), 3-hydroxyloctadecanoylcarnitine (C18-OH), octadecenoylcarnitine (C18:1), 3-hydroxyloctadecenoylcarnitine (C18:1-OH), octadecadienylcarnitine (C18:2). The reference ranges for normal values in MS/MS are presented in Supplementary Table 1.

### Statistical analysis

Participants were categorized into the Anemia group and the Control group based on the presence or absence of gestational anemia. Baseline characteristics, including maternal age, weight, and neonatal birth weight, were compared between the two groups.

The rates of abnormalities in tandem mass spectrometry profiles, AA profiles, and AC profiles were subsequently compared between the two groups. A tandem mass spectrometry abnormality was defined as the presence of at least one abnormal AA or AC indicator in the results. Similarly, an AA profile abnormality was defined as the presence of at least one abnormal AA indicator, and an AC profile abnormality was defined as the presence of at least one abnormal AC indicator. Additionally, individual AA and AC indicators were compared between the two groups.

To identify metabolic pathways associated with differential metabolites influenced by anemia during pregnancy, pathway analysis was conducted using the Pathway Analysis module within the MetaboAnalyst platform. This module integrates pathway enrichment and topology analyses to calculate p-values and pathway impact values, referencing the Small Molecule Pathway Database (SMPDB).

Sensitive analyses were conducted on Normal birth weight infant (NBW) infants and term infants (TI).

The T-test was used for normally distributed continuous variables, the Wilcoxon rank-sum (Mann-Whitney) test was used for non-normally distributed continuous variables, and the χ^2^ test was used for categorical variables. Spearman rank correlation was used to assess correlations between anemia during pregnancy and neonatal metabolic indicators. The *P* < 0.05 was considered to be statistically significant.

## Results

**Fig. 1:**
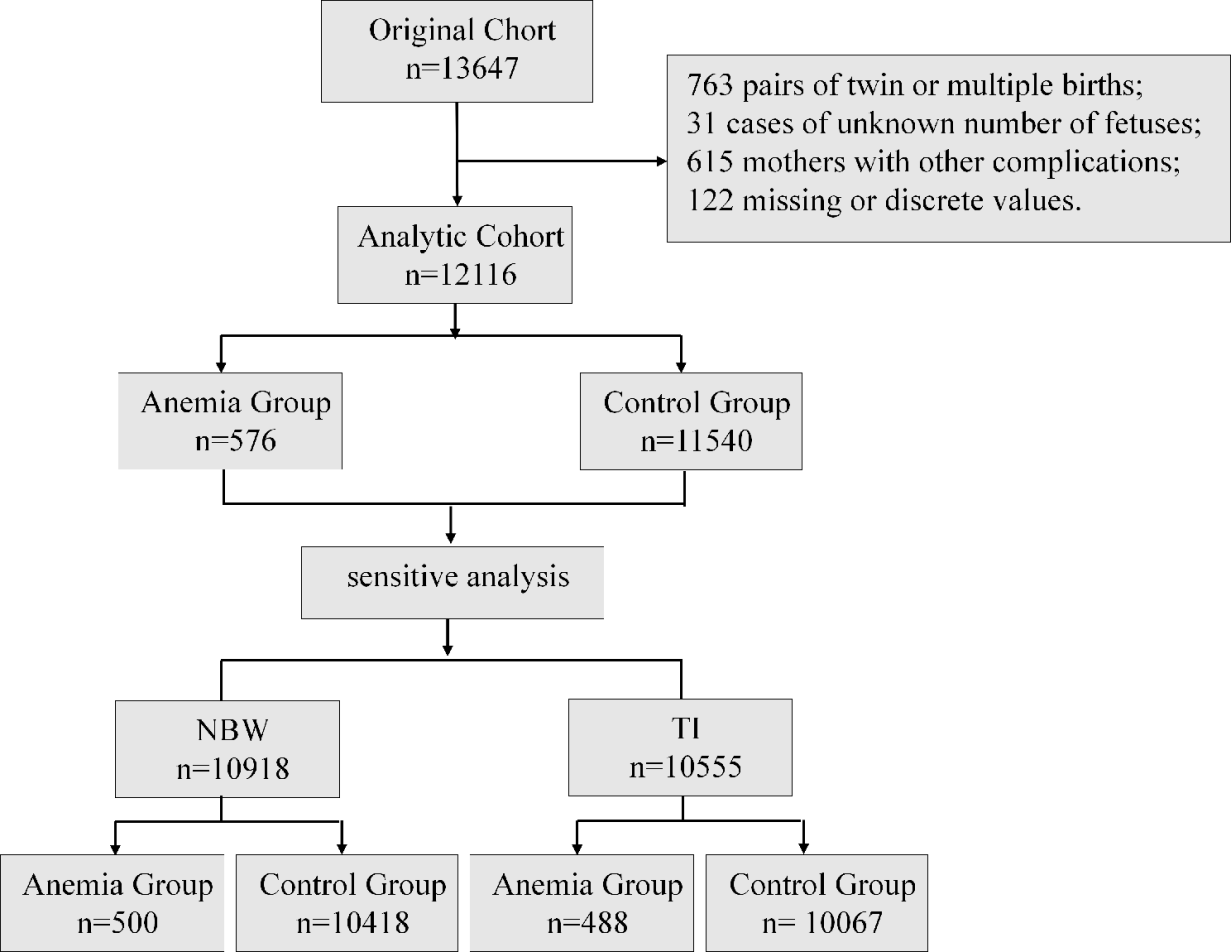
Study Flowchart

A total of 13,647 mother-newborn pairs were initially enrolled in the study. Exclusions included 763 pairs involving twin or multiple births, 31 cases with an unknown number of fetuses, 615 cases with comorbid cardiac, hepatic, or renal diseases, and 122 cases with missing data or discrete values. After these exclusions, 12,116 mother-child pairs were retained for the following analysis. The study flowchart is presented in Fig. 1.

### Characteristics of the participants by Anemia status during pregnancy

The baseline characteristics of the 12,116 mother-newborn pairs included in the study are summarized in Table 1. Among them, 576 pairs were in the Anemia group, while 11,540 pairs in the Control group. No significant differences were observed between the two groups in terms of gestational age, comorbidity with diabetes, hypertension, and hypothyroidism during pregnancy, or newborn sex (*P* > 0.05). Newborns in the Anemia group had a significantly lower birth weight compared to those in the Control group (*P* < 0.05). Additionally, the rates of low birth weight newborns and preterm infants in the Anemia group were significantly higher than in the Control group (*P* < 0.05).

**Table 1:** Characteristics of the participants by Anemia status during pregnancy

	All *n* = 12,116	Anemia Group *n* = 576	Control Group *n* = 11,540	*P* value
**Maternal characteristics**				
Maternal age (years)	33(30,35)	33.01 ± 4.05	32.94 ± 3.91	0.321
Diabetes in pregnancy	2215 (18.28)	95 (16.49)	2120 (18.37)	0.255
Hypertension in pregnancy	1259 (10.39)	59 (10.24)	1200 (10.4)	0.905
Hypothyroidism in pregnancy	1445 (11.93)	54 (9.38)	1391 (12.05)	0.053
Fetal growth restriction	457 (3.77)	20 (3.47)	437 (3.79)	0.699
**Neonatal characteristics**				
Gestational age (weeks)	38.80 ± 1.59	38.25 ± 2.29	38.83 ± 1.54	0.000
Male sex	6250 (51.5)	280 (48.61)	5960 (51.65)	0.155
Female sex	5866 (48.5)	296 (51.39)	5580 (48.35)	0.155
Birth weight (g)	3303.0 ± 469.0	3207.9 ± 569.5	3307.7 ± 462.9	0.001
Low birth weight	520 (4.29)	47 (8.16)	473 (4.10)	0.000
Normal birth weight	10,918 (90.11)	500 (86.81)	10,418 (90.28)	0.006
Macrosomia	678 (5.60)	29 (5.03)	649 (5.62)	0.548
Preterm Infant	689 (5.69)	65 (11.28)	624 (5.41)	0.000

Data are presented as mean± SD, median(interquartile range), or n(%). The T-test was used for normally distributed continuous variables, the Wilcoxon rank-sum (Mann-Whitney) test was used for non-normally distributed continuous variables, and the χ^2^ test was used for categorical variables. The *P* < 0.05 was considered to be statistically significant

### Incidence of neonatal metabolic profile abnormalities

**Fig. 2:**
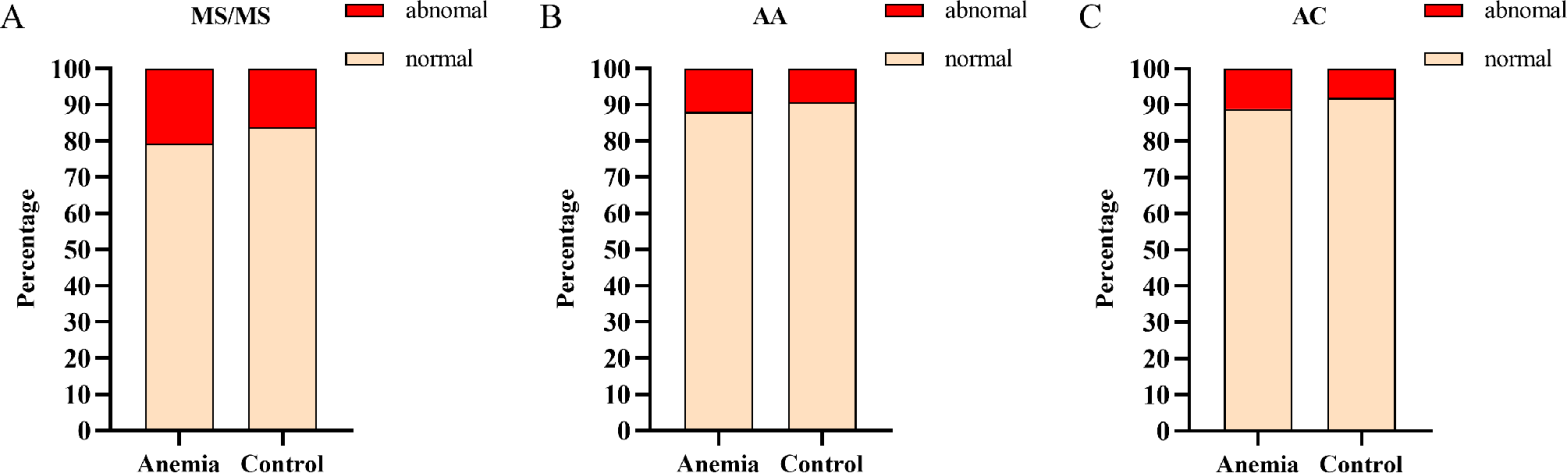
Incidence of Neonatal Metabolic Profile Abnormalities. **A**. The incidence of tandem mass spectrometry abnormalities: Anemia group VS Control group, 20.83%(*n* = 120) VS 16.1%(*n* = 1858), χ^2^ =8.9960, *P <* 0.05;.**B**. The incidence of amino acid (AA) profile abnormalities: Anemia group VS Control group, 11.98%(*n* = 69) VS 9.25%(*n* = 1068), χ^2^ =4.7886, *P <* 0.05; **C**. The incidence of acylcarnitine (AC) profile abnormalities: Anemia group VS Control group, 11.11%(64) VS 8.04%(928), χ^2^ = 6.8763, *P <* 0.05;

The incidence of overall metabolic profile abnormalities was significantly higher in the Anemia group than in the Control group (*P* < 0.05). Similarly, the incidences of AA profile and AC profile abnormalities were also significantly higher in the Anemic group than in the Control group (*P* < 0.05) (Fig. 2).

### Anemia during pregnancy and individual neonatal metabolic indicators

**Fig. 3:**
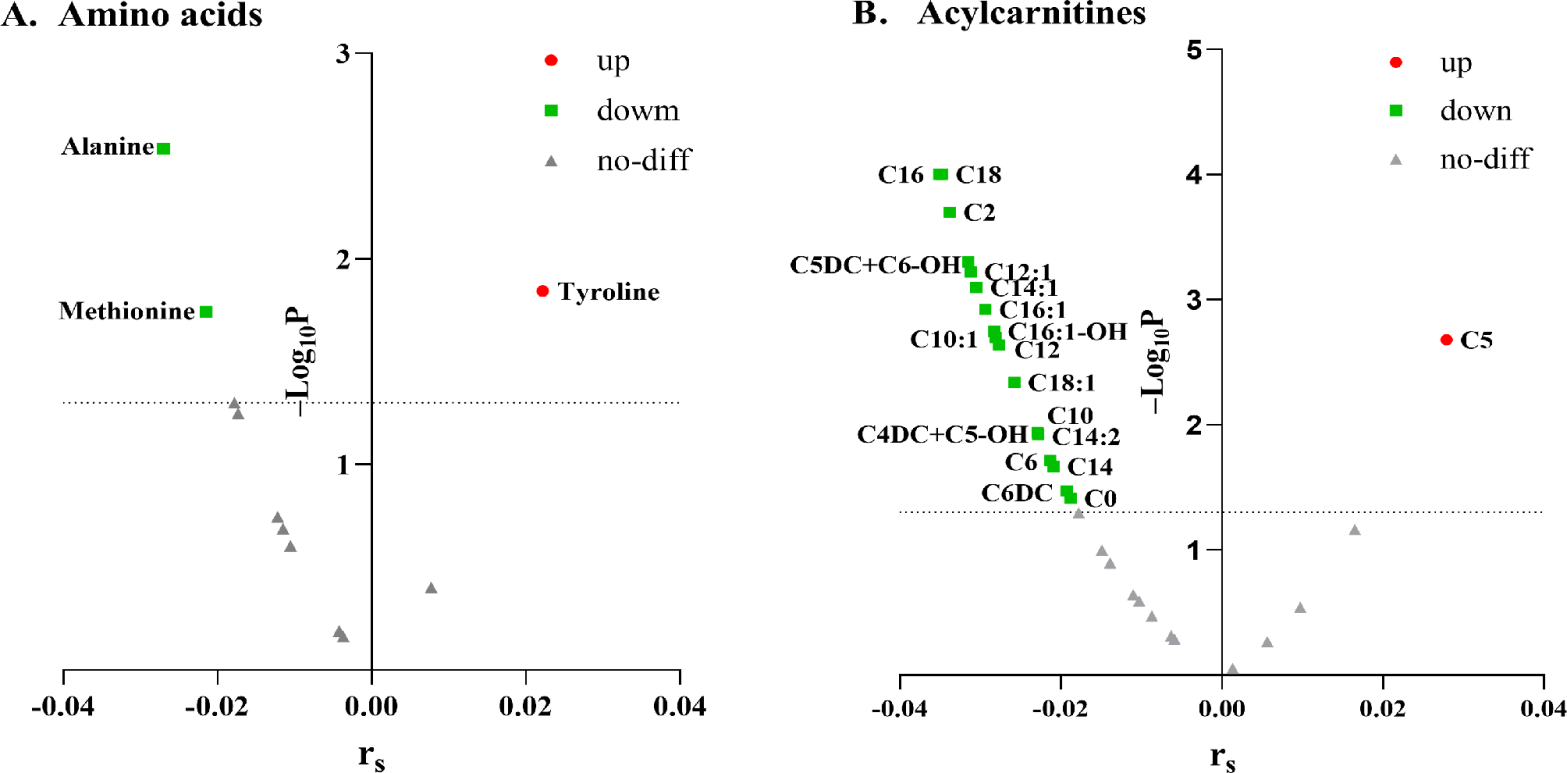
Correlation between anemia during pregnancy and neonatal metabolic indicators. Spearman rank correlation was used to assess correlations between anemia during pregnancy and neonatal metabolic indicators. The *P* < 0.05 was considered to be statistically significant. Red round dots indicate a significant positive correlation, green square dots indicate a significant negative correlation and gray triangular dots indicate no significant correlation

Table 2 presents that 22 neonatal metabolites were significantly different between the Anemia group and the Control group (*P <* 0.05). Alanine and methionine levels were significantly lower in the Anemia group compared to the Control group (*P <* 0.05), while tyrosine levels were significantly higher in the Anemia group than in the Control group (*P <* 0.05). Notably, the majority of ACs, including C0, C2, C4DC + C5-OH, C5DC + C6-OH, C6, C6DC, C10, C10:1, C12, C12:1, C14, C14:1, C14:2, C16, C16:1, C16:1OH, C18, and C18:1, were significantly lower in the Anemia group compared to the Control group (*P <* 0.05). Conversely, C5 levels were significantly higher in the Anemia group than in the Control group (*P <* 0.05). Spearman correlation analysis further confirmed a significant relationship between anemia during pregnancy and certain AAs and ACs, aligning with the above results (Fig. 3).

**Table 2:** Comparison of metabolic indicators between the Anemia group and control group

Metabolic indicators	Control	Anemia	*P* value
Alanine	326.235 (266.99, 393.01)	314.65 (255.56, 379.6)	0.003
Arginine	15.11 (9.2, 22.835)	13.865 (8.045, 22.945)	0.057
Citrulline	13 (10.77, 15.805)	12.695 (10.57, 15.525)	0.051
Glycine	417.61 (343.92, 505.78)	415.23 (347.775, 496.855)	0.645
Leucine + sioleucine +hydroxyproline	155.63 (131.605, 184.58)	156.3 (133.62, 185.965)	0.397
Methionine	24.85 (20.58, 29.645)	23.92 (19.46, 29.4 )	0.018
Ornithine	125.155 (100.88, 158.325)	123.075 (98.925, 154.59)	0.249
phenylalanine	51.02 (45.105, 57.9)	51.08 (45.2, 57.745)	0.687
Proline	173.03 (147.295, 205.735)	169.45 (144.415, 202.56 )	0.180
Tyrosine	104.855 (83.86, 131.8)	108.155 ( 83.535, 142.28)	0.014
Valine	133.675 (112.455, 158.78)	132.77 (109.845, 157.075)	0.206
C0	21.035 (16.85, 25.87)	20.335 (16.095, 25.395)	0.039
C2	16.88 (13.25, 21.635)	16.055 (12.265, 20.62)	0.000
C3	1.275 (0.97, 1.67)	1.3 (0.95, 1.66)	0.883
C3DC + C4-OH	0.1 (0 0.07, 0 0.14)	0.09 (0.07, 0.14)	0.051
C4	0.18 (0.15, 0 0.22)	0 0.18 (0.15, 0.21)	0.487
C4DC + C5-OH	0.18 (0 0.15, 0 0.22)	0.17 (0.14, 0.205)	0.012
C5	0.09 (0.08, 0.12)	0.1 (0.08, 0 0.13)	0.002
C5:1	0.01 (0, 0.01)	0.01 (0, 0.01)	0.069
C5DC + C6-OH	0.1 (0.08, 0 0.12)	0.09 (0.07, 0.12)	0.001
C6	0.04 (0 0.03, 0.04)	0.03 (0.03, 0.04)	0.019
C6DC	0 0.11 (0.09, 0.15)	0 0.11 (0.08, 0.14)	0.034
C8	0.07 (0.05, 0.09)	0.07 (0.05, 0.09)	0.516
SA	0.63 (0.52, 0.74)	0.62 ( 0.5, 0.74 )	0.338
C10	0.07 (0.05, 0.1)	0 0.07 (0.05, 0.09)	0.012
C10:1	0.06 (0.05, 0.08)	0.06 (0.04, 0.07)	0.002
C10:2	0.01 (0.01, 0.01)	0.01 (0.01, 0.01)	0.539
C12	0.06 (0.05, 0.08)	0.06 (0.04, 0 0.08)	0.002
C12:1	0.04 (0.03, 0.07)	0.04 (0.02, 0.06)	0.001
C14	0.15 (0.11, 0.18)	0.14 (0.11, 0.18)	0.022
C14-OH	0.01 (0.01, 0.01)	0.01 (0.01, 0.01)	0.228
C14:1	0.07 (0.05, 0.09)	0.06 (0.04, 0.08)	0.001
C14:2	0.02 (0.01, 0.04)	0.02 (0.01, 0.02)	0.012
C16	2.34 (1.61, 3.17)	2.11 (1.4, 2.94)	0.000
C16-OH	0.01 (0.01, 0.02)	0.01 (0.01, 0.02))	0.100
C16:1	0.11 (0.07, 0.17)	0.1 (0.06, 0.16)	0.001
C16:1-OH	0.03 (0.02, 0.04)	0.03 (0.02, 0.03)	0.002
C18	0.68 (0.52, 0.86)	0.65 (0.49, 0.82)	0.000
C18-OH	0.01 (0.01, 0.01)	0.01 (0.01, 0.01)	0.256
C18:1	1.26 (1.01, 1.55)	1.21 (0.965, 1.148)	0.005
C18:1-OH	0.01 (0.01, 0.02)	0.01 (0.01, 0.02)	0.127
C18:2	0.23 (0.17, 0.31)	0.24 (0.17, 0.32)	0.288

The data were presented as median (interquartile range). The Wilcoxon rank-sum (Mann-Whitney) test was used to compare differences in metabolic indicators between the Anemia group and the Control group. The *P* < 0.05 was considered to be statistically significant

### Metabolic pathways associated with Anemia during pregnancy

**Fig. 4:**
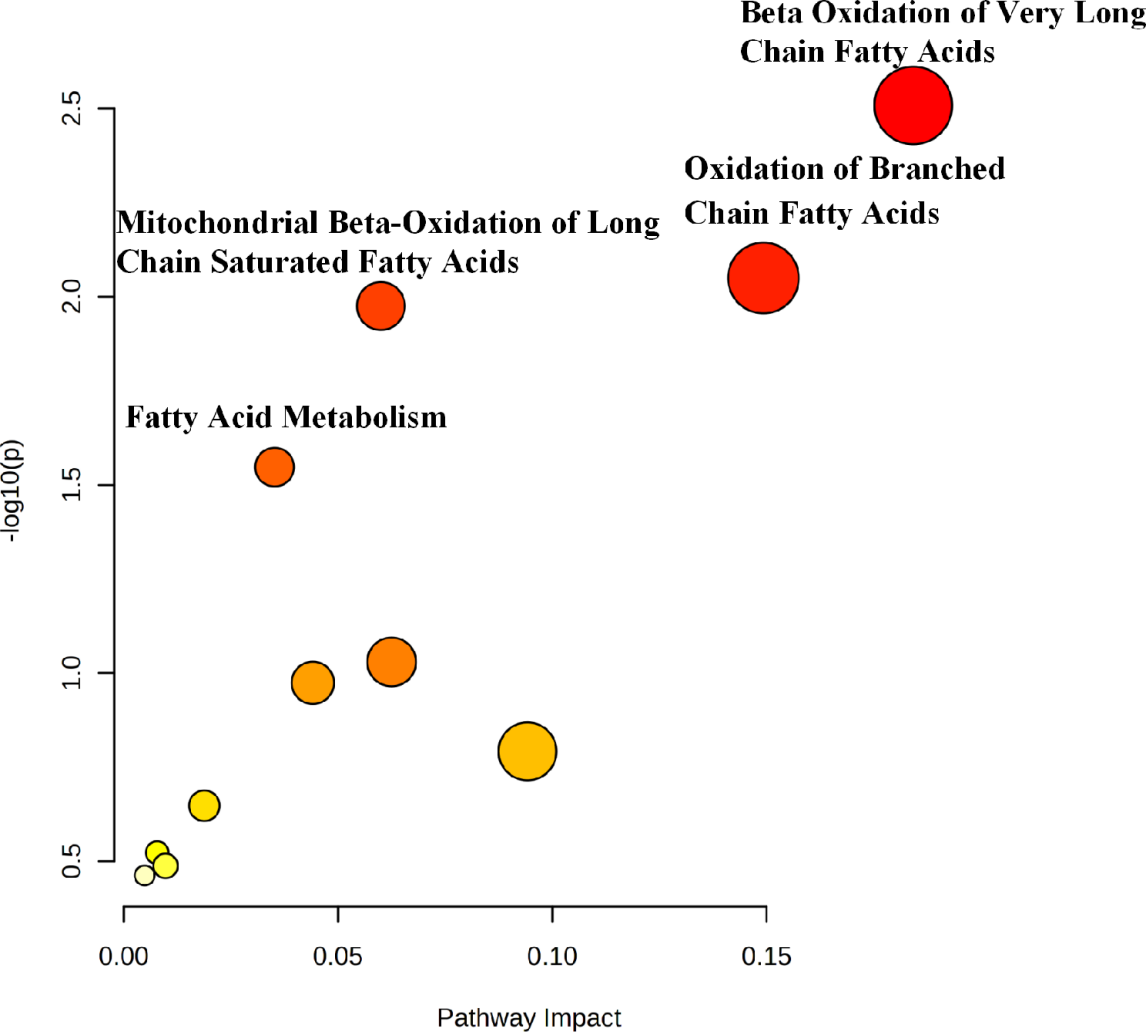
Pathway analysis. Pathway enrichment and topology analyses identified several neonatal metabolic pathways associated with anemia during pregnancy: beta-oxidation of very long-chain fatty acids, oxidation of branched-chain fatty acids, mitochondrial beta-oxidation of long-chain saturated fatty acids, and fatty acid metabolism. *P <* 0.05

Pathway enrichment and topology analyses further identified several metabolic pathways associated with anemia during pregnancy. Reduced AC levels were associated with beta-oxidation of very long-chain fatty acids, oxidation of branched-chain fatty acids, mitochondrial beta-oxidation of long-chain saturated fatty acids, and fatty acid metabolism (Fig. 4), all of which were integral to FAO. However, due to the limited range of metabolites measured, no significant findings were observed for pathways related to methionine, alanine, and tyrosine metabolism.

### Sensitivity analysis

**Fig. 5:**
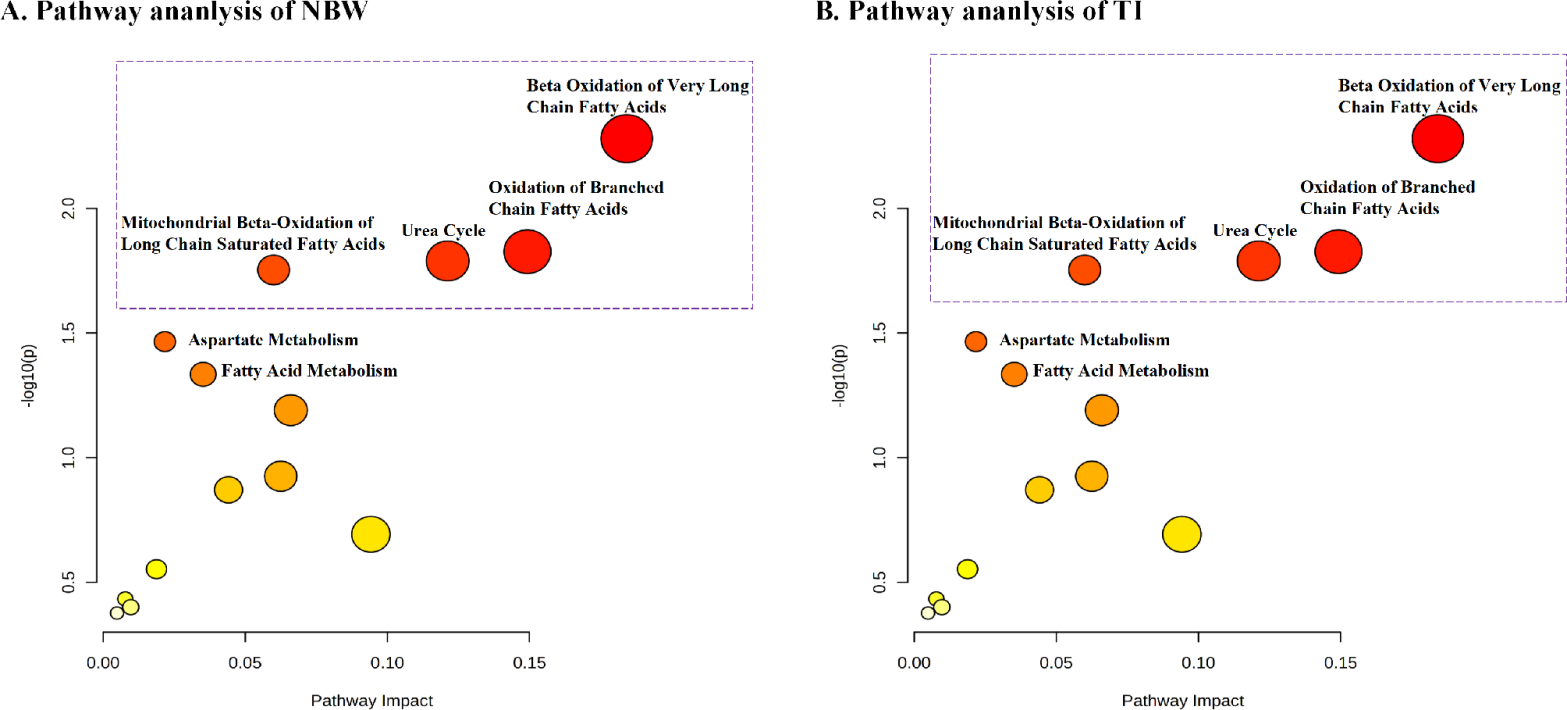
Sensitive analysis. (**A**) Pathway enrichment and topology analyses indicate neonatal metabolic pathways in normal birth weight(NBW) infants associated with anemia during pregnancy: beta-oxidation of very long-chain fatty acids, oxidation of branched-chain fatty acids, mitochondrial beta-oxidation of long-chain saturated fatty acids, fatty acid metabolism, aspartate metabolism and the urea cycle. (**B**) Pathway enrichment and topology analyses indicate neonatal metabolic pathways term infants(TI) associated with anemia during pregnancy: beta-oxidation of very long-chain fatty acids, oxidation of branched-chain fatty acids, mitochondrial beta-oxidation of long-chain saturated fatty acids, fatty acid metabolism, aspartate metabolism, and the urea cycle

Given the significant differences in neonatal birth weight and preterm birth rates between the Anemia and Control groups, sensitivity analyses were conducted on two sub-cohorts: NBW infants and TIs. The differences in alanine, methionine, tyrosine, and most ACs between the two groups persisted in both two sub-cohorts. In NBW infants, the decreased AC indicators in the Anemia group included C0, C2, C3DC + C5-OH, C4DC + C5-OH, C5DC + C6-OH, C6, C10:1, C12:1, C14:1, and C14:2. For TIs, the reduced AC indicators included C0, C2, C4DC + C5-OH, C5DC + C6-OH, C6, C10:1, C12:1, C14:1, C16, C16:1, and C18(Supplementary Tables 2, 3). Pathway analysis for both sub-cohorts revealed associations with the beta-oxidation of very long-chain fatty acids, oxidation of branched-chain fatty acids, mitochondrial beta-oxidation of long-chain saturated fatty acids, and fatty acid metabolism (Fig. 5). These findings aligned with the results of the whole cohort study.

No significant differences in C5 between the Anemia group and Control group among NBW infants and TIs. Additionally, in both NBW infants and TI, a significant reduction in citrulline and arginine levels was observed in the Anemia group (Supplementary Tables 2, 3). Pathway analysis indicated that these reductions were associated with aspartate metabolism and the urea cycle (Fig. 5).

## Discussion

Anemia during pregnancy is a prevalent complication with significant health implications for both mothers and their offspring. However, its impact on neonatal metabolic patterns remains inadequately studied. To our knowledge, this study is the first to systematically examine the association between maternal anemia during pregnancy and neonatal metabolic profiles, providing novel insights into the metabolic consequences of maternal anemia during pregnancy.

Since the pregnant women recruited in this study were those in their third trimester, the incidence of anemia during pregnancy was only 5% (576/12116), which is lower than the rates reported in the literature [3]. Previous studies have shown that pregnant women with anemia are at a higher risk of delivering preterm or low birth weight (LBW) infants [4, 6]. Recent researches suggest that iron and folic acid supplementation during pregnancy may increase neonatal birth weight [20] and reduce the incidence of LBW [21], but has no significant effect on preterm birth or neonatal mortality. In our study, the anemia group had a persistently higher incidence of preterm birth and LBW compared to the non-anemia group. However, we were unable to determine whether these pregnant women received treatment or how treatment influenced neonatal outcomes. Future research is needed to further investigate these aspects.

More importantly, our study revealed a higher incidence of metabolic abnormalities in Anemia group. Specially, we observed significant reductions in multiple AC species, along with lower methionine and alanine levels and elevated tyrosine levels. These metabolic alterations persisted in sensitivity analyses restricted to normal birth weight and term infants, reinforcing the robustness of our findings. Although the differences in individual indicators were not large, these metabolic changes may result from multiple factors acting together. Indicators with minor differences may represent just one component of a broader network of influencing factors.

### Increased incidence of neonatal metabolic profile abnormalities in Anemia group

In this investigation, we observed a higher incidence of abnormalities in the overall neonatal metabolic profile, as well as in AA and AC profiles, in the Anemia group compared to the Control group. This phenomenon may contribute to an increased false-positive rate in newborn screening, potentially leading to a greater social burden. Our findings provide valuable insights into the possible reasons behind false-positive results in newborn screening and highlight the impact of maternal anemia on metabolic screening outcomes. However, due to the low incidence of abnormalities in individual biomarkers, further statistical analysis was not feasible at this stage. To mitigate this limitation and strengthen the reliability of our findings, future studies will aim to expand the sample size.

### Decreased acylcarnitine levels in Anemia group

Nearly all ACs, including C0, C2, C4DC + C5-OH, C5DC + C6-OH, C6, C6DC, C10, C10:1, C12, C12:1, C14, C14:1, C14:2, C16, C16:1, C16:1OH, C18, and C18:1, were significantly lower in the Anemia group compared to the Control group. Pathway analysis indicated that these ACs are involved in key metabolic processes, including the β-oxidation of very long-chain fatty acids, the oxidation of branched-chain fatty acids, mitochondrial β-oxidation of long-chain saturated fatty acids, and overall fatty acid metabolism. These pathways are vital for FAO, and their robustness was further confirmed in sensitivity analyses in NBW infants and TIs. FAO is a critical energy production pathway in mammals, particularly during fasting, when fatty acids become the primary substrate for energy production in the liver, cardiac muscle, and skeletal muscle [22]. Given its fundamental role in energy metabolism, we hypothesize that FAO suppression in neonates born to anemic mothers may increase the risk of hypoglycemia, metabolic inflexibility, and long-term metabolic disturbances, including insulin resistance, lipid accumulation, and cardiac inefficiency [23, 24]. Furthermore, FAO impairment may disrupt hepatic metabolism, increasing susceptibility to fatty liver disease [25]. Further studies are needed to assess the long-term metabolic impact of early-life FAO suppression.

However, the specific mechanisms by which maternal anemia impacts FAO need further study. L-carnitine supplementation has been reported to improve hemoglobin levels in dialysis patients [26–29], suggesting a potential interaction between carnitine and anemia. We hypothesize two potential mechanisms for these observations. Firstly, anemia during pregnancy may impair the absorption or synthesis of free carnitine in newborns. Carnitine plays a crucial role in energy metabolism by facilitating the transport of long-chain fatty acids into mitochondria for subsequent β-oxidation. A reduction in free carnitine levels would subsequently decrease the levels of ACs. Carnitine homeostasis depends on dietary absorption, endogenous biosynthesis, and renal reabsorption, with the latter mediated by the organic cation transporter novel family member 2 (OCTN2) [30]. We hypothesize that maternal anemia may impair OCTN2 function, directly reducing neonatal OCTN2 activity and carnitine absorption or indirectly affecting neonatal carnitine levels by compromising maternal OCTN2 function. The latter may reduce maternal carnitine absorption and retention subsequently influencing neonatal C0 concentrations and carnitine homeostasis [31]. Moreover, both anemia and low carnitine levels during pregnancy may result from insufficient dietary intake, particularly low meat consumption, which can lead to inadequate nutrient supply [32, 33]. Therefore, poor maternal nutrition may serve as a common underlying factor for both gestational anemia and low carnitine levels, potentially leading to reduced neonatal C0 levels. Secondly, endogenous carnitine synthesis requires adequate levels of lysine and methionine. Methionine levels were significantly lower in the newborns from the Animia group, potentially contributing to reduced endogenous carnitine synthesis. This combined disruption in both maternal and endogenous carnitine sources may explain the observed reductions in ACs in newborns from the Anemia group.

Interestingly, among the AC profiles, only C5 levels were significantly elevated in the Anemic group. Isovalerylcarnitine (C5) is formed through the conjugation of isovaleryl-CoA with free carnitine. Isovaleryl-CoA serves as metabolic substrate for isovaleryl-CoA dehydrogenase, which catalyzes its conversion into β-methylcrotonyl-CoA [34, 35]. Quantification of C5 in dried blood spots is commonly used for screening isovaleric acidemia (IVA). However, tandem mass spectrometry profiling has a high false-positive rate for C5, often due to interference from pivaloylcarnitine, an isobaric compound found in substances like certain antibiotics and emollients [36]. These confounders may falsely elevate C5 levels during newborn screening. Furthermore, studies have reported higher C5 levels in small-for-gestational-age (SGA) infants [37] This may be attributed to the immaturity of isovaleryl-CoA dehydrogenase function in SGA infants. To address these potential confounders, we conducted a sensitive analysis and found no significant differences in C5 between the Anemia group and Control group among NBW infants and TIs. This suggests that the observed elevation of C5 in the Anemia group may be influenced by birth weight and gestational age, rather than reflecting a true increase in C5 levels.

### Decreased methionine, Alanine, and increased tyrosine in Anemia group

Methionine, a sulfur-containing essential amino acid, serves as a key precursor for succinyl-CoA, homocysteine, cysteine, creatine, and carnitine. Emerging research highlights its vital role in modulating metabolic pathways, innate immunity, and digestive function in mammals [38]. A reduction in methionine levels may disrupt these physiological processes, potentially leading to broader systemic imbalances. As an essential AA, methionine is primarily obtained from the diet but can also be recycled via the methionine salvage pathway (MSP). Diets predominantly based on plant sources are often associated with deficiencies in protein, iron, and vitamin B12, all of which are linked to anemia during pregnancy [39, 40]. The MSP plays a pivotal role in regenerating methionine from 5’-methylthioadenosine (MTA) through six enzymatic steps under aerobic conditions. Evidence from *Schizosaccharomyces pombe* indicates that the genes involved in the third and fifth steps of MSP ( mug14 + and adi1+), are repressed under low-iron conditions [41]. Consequently, maternal anemia might contribute to reduced methionine levels through both dietary deficiencies and iron-dependent suppression of the MSP.

Alanine is primarily synthesized endogenously in skeletal muscle. Its synthesis and release are heightened during periods of active glycolysis, such as fasting, when glycolysis and glycogenolysis are activated in muscle tissue [42–44]. Previous studies have demonstrated a positive correlation between alanine levels in fetuses or newborns and maternal gestational health [45–47]. Elevated tyrosine levels were also observed in the Anemia group. Tyrosine serves as a precursor for catecholamines (e.g., norepinephrine, epinephrine, dopamine) and melanins (e.g., eumelanins and pheomelanins) [48]. Although no significant results were detected in the pathway analysis for alanine- and tyrosine-related metabolic pathways due to the limited range of measured metabolites, these pathways remain of interest for further investigation. Additionally, the potential health implications of alterations in these metabolic pathways warrant further research.

While citrulline and arginine levels were not significantly different between the Anemia group and Control group in the whole cohort, their significant reduction in the Anemia group among NBW infants and TIs in sensitivity analyses suggests potential disturbances in aspartate metabolism and the urea cycle. This finding highlights the need for further research to elucidate the mechanisms underlying these metabolic changes.

### Limitations

This study has several limitations. Firstly, it only included data on anemia during late pregnancy, without distinguishing the severity of anemia. Secondly, the neonatal metabolic data were derived from newborn screening programs, which included only 11 AAs and 31 ACs. This limited scope may not fully capture the broader spectrum of neonatal metabolism and constrain the results of the pathway analysis. Thirdly, information on anemia in newborns and metabolic profiles of mothers was not collected, potentially introducing confounding factors into the analysis.

## Conclusions

Maternal anemia during pregnancy is significantly associated with alterations in neonatal metabolic profiles, particularly in fatty acid beta-oxidation and related pathways. These findings highlight the potential metabolic consequences of gestational anemia and provide insights into its role in adverse neonatal outcomes and abnormal newborn screening results. While our study contributes to understanding the metabolic impact of maternal anemia, limitations such as sample size and the lack of longitudinal follow-up should be addressed in future research. Further investigations are needed to determine whether these metabolic alterations have long-term clinical implications and whether targeted nutritional interventions during pregnancy could mitigate these effects.

## Electronic supplementary material

Below is the link to the electronic supplementary material.

Supplementary Material 1

## Data Availability

Please contact the corresponding author for data requests.

## Abbreviations

AA: Amino acid
AC: Acylcarnitine
FAO: Fatty acid oxidation
MS/MS: Tandem mass spectrometry
MSP: Methionine salvage pathway
MTA 5': Methylthioadenosine
NBW: Normal birth weight
OCTN2: Organic cation transporter novel family member 2
SGA: Small-for-gestational-age
SMPDB: Small Molecule Pathway Database
TI: Term infants
WHO: World Health Organization
