# Supplementary material for: Impact of maternal anemia during pregnancy on neonatal metabolic profiles: evidence from the Beijing Birth Cohort Study
Source: BMC Pregnancy Childbirth. 2025 Apr 26;25:506. doi: 10.1186/s12884-025-07626-9 (PMC12032785; doi:10.1186/s12884-025-07626-9)
Supplement: Supplementary file 1 — Supplementary Material 1 [file 12884_2025_7626_MOESM1_ESM.doc]

**Supplementary material**

**Catalogue**

Supplementary Table 1. The reference ranges for normal values in tandem mass spectrometry (MS/MS)

Supplementary Table 2. The Correlation Between Anemia During Pregnancy and Neonatal Metabolic Indicators in Normal Birthweight (NBW) Infants

Supplementary Table 3. The Correlation Between Anemia During Pregnancy and Neonatal Metabolic Indicators in Term Infants (TI)

Supplementary Table 1. The reference ranges for normal values in MS/MS

| Indicators in MS/MS | | Reference ranges (μmol/L) |
| --- | --- | --- |
| Amino acids | Alanine | 170-740 |
|  | Arginine | 2-60 |
|  | Citrulline | 7.6-50 |
|  | Glycine | 210-1200 |
|  | Leucine+sioleucine+hydroxyproline | 90-350 |
|  | Methionine | 10-60 |
|  | Ornithine | 60-400 |
|  | phenylalanine | 30-110 |
|  | Proline | 110-480 |
|  | Tyroline | 40-400 |
|  | Valine | 60-280 |
| Acylcarnitines | C0 | 10-60 |
|  | C2 | 7-60 |
|  | C3 | 0.5-5.7 |
|  | C3DC+C4-OH | 0-0.45 |
|  | C4 | 0.1-0.5 |
|  | C4DC+C5-OH | 0-0.5 |
|  | C5 | 0-0.4 |
|  | C5:1 | 0-0.08 |
|  | C5DC+C6-OH | 0.05-0.35 |
|  | C6 | 0-0.18 |
|  | C6DC | 0-0.4 |
|  | C8 | 0-0.3 |
|  | SA | 0.3-1 |
|  | C10 | 0-0.4 |
|  | C10:1 | 0-0.2 |
|  | C10:2 | 0-0.09 |
|  | C12 | 0-0.45 |
|  | C12:1 | 0-0.3 |
|  | C14 | 0-0.5 |
|  | C14-OH | 0-0.07 |
|  | C14:1 | 0-0.45 |
|  | C14:2 | 0-0.08 |
|  | C16 | 0.5-7.4 |
|  | C16-OH | 0-0.07 |
|  | C16:1 | 0-0.5 |
|  | C16:1-OH | 0-0.09 |
|  | C18 | 0.2-2.1 |
|  | C18-OH | 0-0.05 |
|  | C18:1 | 0.5-3.5 |
|  | C18:1-OH | 0-0.7 |
|  | C18:2 | 0.05-0.85 |

Supplementary Table 2. The Correlation Between Anemia During Pregnancy and Neonatal Metabolic Indicators in NBW Infants

| Metabolic indicators | rs | *P* value |
| --- | --- | --- |
| Alanine | -0.0225 | 0.019 |
| Arginine | -0.0257 | 0.007 |
| Citrulline | -0.0284 | 0.003 |
| Glycine | 0.0005 | 0.956 |
| Leucine+sioleucine+hydroxyproline | 0.0045 | 0.641 |
| Methionine | -0.0323 | 0.001 |
| Ornithine | -0.0110 | 0.250 |
| phenylalanine | -0.0089 | 0.350 |
| Proline | -0.0063 | 0.511 |
| Tyroline | 0.0302 | 0.002 |
| Valine | -0.0095 | 0.321 |
| C0 | -0.0253 | 0.008 |
| C2 | -0.0343 | 0.000 |
| C3 | 0.0026 | 0.788 |
| C3DC+C4-OH | -0.0190 | 0.048 |
| C4 | -0.0083 | 0.387 |
| C4DC+C5-OH | -0.0259 | 0.007 |
| C5 | 0.0158 | 0.099 |
| C5:1 | 0.0102 | 0.286 |
| C5DC+C6-OH | -0.0301 | 0.002 |
| C6 | -0.0270 | 0.005 |
| C6DC | -0.0182 | 0.058 |
| C8 | -0.0077 | 0.423 |
| SA | -0.0077 | 0.418 |
| C10 | -0.0179 | 0.062 |
| C10:1 | -0.0238 | 0.013 |
| C10:2 | 0.0019 | 0.844 |
| C12 | -0.0174 | 0.069 |
| C12:1 | -0.0233 | 0.015 |
| C14 | -0.0109 | 0.256 |
| C14-OH | -0.0043 | 0.653 |
| C14:1 | -0.0217 | 0.023 |
| C14:2 | -0.0192 | 0.045 |
| C16 | -0.0232 | 0.015 |
| C16-OH | -0.0136 | 0.155 |
| C16:1 | -0.0195 | 0.041 |
| C16:1-OH | -0.0175 | 0.067 |
| C18 | -0.0239 | 0.013 |
| C18-OH | -0.0031 | 0.747 |
| C18:1 | -0.0185 | 0.054 |
| C18:1-OH | -0.0069 | 0.470 |
| C18:2 | 0.0022 | 0.822 |

Spearman rank correlation was used to assess correlations between anemia during pregnancy and neonatal metabolic indicators. The *P* < 0.05 was considered to be statistically significant.

Supplementary Table 3. The Correlation Between Anemia During Pregnancy and Neonatal Metabolic Indicators in TI

| Metabolic indicators | rs | *p* value |
| --- | --- | --- |
| Alanine | -0.0176 | 0.071 |
| Arginine | -0.0207 | 0.033 |
| Citrulline | -0.0243 | 0.017 |
| Glycine | 0.0029 | 0.765 |
| Leucine+sioleucine+hydroxyproline | 0.0113 | 0.245 |
| Methionine | -0.0268 | 0.006 |
| Ornithine | -0.0065 | 0.501 |
| phenylalanine | -0.0045 | 0.647 |
| Proline | -0.0005 | 0.958 |
| Tyroline | 0.0303 | 0.002 |
| Valine | 0.0004 | 0.964 |
| C0 | -0.0207 | 0.034 |
| C2 | -0.0298 | 0.002 |
| C3 | 0.0061 | 0.532 |
| C3DC+C4-OH | -0.0149 | 0.125 |
| C4 | -0.0109 | 0.264 |
| C4DC+C5-OH | -0.0206 | 0.034 |
| C5 | 0.0159 | 0.103 |
| C5:1 | 0.0114 | 0.242 |
| C5DC+C6-OH | -0.0325 | 0.001 |
| C6 | -0.0287 | 0.003 |
| C6DC | -0.0150 | 0.123 |
| C8 | -0.0082 | 0.400 |
| SA | -0.0059 | 0.544 |
| C10 | -0.0178 | 0.067 |
| C10:1 | -0.0227 | 0.020 |
| C10:2 | 0.0054 | 0.577 |
| C12 | -0.0149 | 0.126 |
| C12:1 | -0.0236 | 0.016 |
| C14 | -0.0054 | 0.579 |
| C14-OH | -0.0037 | 0.705 |
| C14:1 | -0.0223 | 0.022 |
| C14:2 | -0.0158 | 0.105 |
| C16 | -0.0209 | 0.032 |
| C16-OH | -0.0096 | 0.322 |
| C16:1 | -0.0200 | 0.040 |
| C16:1-OH | -0.0158 | 0.105 |
| C18 | -0.0206 | 0.034 |
| C18-OH | 0.0000 | 0.996 |
| C18:1 | -0.0141 | 0.147 |
| C18:1-OH | -0.0009 | 0.929 |
| C18:2 | 0.0096 | 0.326 |

Spearman rank correlation was used to assess correlations between anemia during pregnancy and neonatal metabolic indicators. The *P* < 0.05 was considered to be statistically significant.
